# Supplementary material for: End Sequence Analysis Toolkit (ESAT) expands the extractable information from single-cell RNA-seq data
Source: Genome Res. 2016 Oct;26(10):1397–410. doi: 10.1101/gr.207902.116 (PMC5052061; doi:10.1101/gr.207902.116)
Supplement: Supplemental Material [file supp_gr.207902.116_Supp_Materials.tar.gz › ESAT-esat_v0.1/doc/html/classjaligner_1_1_markups.html]

ESAT: jaligner.Markups Class Reference


ESAT


- Main Page
- Related Pages
- Packages
- Classes
- Files

- Class List
- Class Index
- Class Hierarchy
- Class Members

All Classes Namespaces Files Functions Variables Enumerator Pages

- jaligner
- Markups

Static Public Attributes |
List of all members

jaligner.Markups Class Referenceabstract

|  |  |
| --- | --- |
| Static Public Attributes | |
| static final char | IDENTITY = '\|' |
|  | |
| static final char | SIMILARITY = ':' |
|  | |
| static final char | GAP = ' ' |
|  | |
| static final char | MISMATCH = '.' |
|  | |

## Detailed Description

Markups line characters.

Author
:   Ahmed Moustafa (ahmed.nosp@m.@use.nosp@m.rs.sf.nosp@m..net)

## Member Data Documentation

final char jaligner.Markups.GAP = ' '

static

Markup line gap character

final char jaligner.Markups.IDENTITY = '|'

static

Markup line identity character

final char jaligner.Markups.MISMATCH = '.'

static

Markup line mismatch character

final char jaligner.Markups.SIMILARITY = ':'

static

Markup line similarity character

---

The documentation for this class was generated from the following file:

- java/jaligner/Markups.java


---

Generated on Fri Aug 15 2014 15:11:05 for ESAT by  

 1.8.7
